# 

**Published:** 1809-11

**Authors:** 


### CORREPONDENCE.

We are very sensible of the severity of Dr. R's pen, as we have had occasion to remark: but we cannot insert anonymous observations on a gentleman who honours us with his name.

*Observator's* paper has not passed unnoticed. We hope however he will recollect how frequently the subject of chemist-apothecaries has been before the public. With him we sincerely regret that a well-educated and meritorious branch of the profession should be so ill rewarded. It is our intention to take up the subject hereafter more at large, when we shall avail ourselves of Observator's remarks in addition to our own.

We are obliged to Dr. Walker for his remarks; but the disease of brenning has been so often referred to, that we fear our Correspondents will be little interested in it.

We are much obliged to Verax for his communication, nor should the want of a real signature have prevented its insertion; but we wish him to remark, that in our present Number he will find it admitted that the remedy is not always successful, and also that the objections he makes to former trials are obviated in these last.—We are, however, thankful for his favour, and if he will shorten it, or permit us to do so, it shall be inserted in a future Number.

Since writing the first paragraph in this "Correspondence," we have received a letter from a Gentleman, who signs himself an Old Correspondent. We can only offer the same answer as the above to a communication in many respects similar.

